# Circular RNAs in Muscle Function and Disease

**DOI:** 10.3390/ijms19113454

**Published:** 2018-11-03

**Authors:** Simona Greco, Beatrice Cardinali, Germana Falcone, Fabio Martelli

**Affiliations:** 1 Molecular Cardiology Laboratory, IRCCS-Policlinico San Donato, San Donato Milanese, 20097 Milan, Italy; simona.greco@grupposandonato.it; 2 Institute of Cell Biology and Neurobiology, National Research Council, Monterotondo, 00015 Rome, Italy; beatrice.cardinali@cnr.it

**Keywords:** circular RNAs, skeletal muscle, cardiac muscle, muscle disease

## Abstract

Circular RNAs (circRNAs) are a class of RNA produced during pre-mRNA splicing that are emerging as new members of the gene regulatory network. In addition to being spliced in a linear fashion, exons of pre-mRNAs can be circularized by use of the 3′ acceptor splice site of upstream exons, leading to the formation of circular RNA species. In this way, genetic information can be re-organized, increasing gene expression potential. Expression of circRNAs is developmentally regulated, tissue and cell-type specific, and shared across eukaryotes. The importance of circRNAs in gene regulation is now beginning to be recognized and some putative functions have been assigned to them, such as the sequestration of microRNAs or proteins, the modulation of transcription, the interference with splicing, and translation of small proteins. In accordance with an important role in normal cell biology, circRNA deregulation has been reported to be associated with diseases. Recent evidence demonstrated that circRNAs are highly expressed in striated muscle tissue, both skeletal and cardiac, that is also one of the body tissue showing the highest levels of alternative splicing. Moreover, initial studies revealed altered circRNA expression in diseases involving striated muscle, suggesting important functions of these molecules in the pathogenetic mechanisms of both heart and skeletal muscle diseases. The recent findings in this field will be described and discussed.

## 1. Introduction

Although skeletal and cardiac muscles originate from different mesodermal precursors in the embryo, they are both designed to generate force and movement in the body. They are collectively called striated muscles because the filaments of actin and myosin that power their contraction are organized into repeating arrays, called sarcomeres, that have a striated microscopic appearance [1]. While some signaling pathways are important to coordinate the proliferation and expansion of myogenic precursors, as well as proper patterning and differentiation common to skeletal and cardiac muscles, others are unique to each muscle type [1,2]. Environmental signals have an important role in the regeneration and growth of striated muscle tissue postnatally and in muscle aging [3,4]. Both skeletal and cardiac muscles undergo degeneration in a number of muscle disorders. Over the past decade, several studies have shown that a variety of newly discovered or re-discovered classes of non-coding RNAs are associated with striated muscle tissue development and disease [5,6]. In this review, we will focus on circular RNAs and explore the state of knowledge on this class of transcripts in regulating gene expression in healthy and diseased striated muscles.

## 2. Definition and Analysis of Circular RNAs

CircRNAs are a class of RNA characterized by a closed ring structure without 3′ and 5′ ends, that are generated by splicing events occurring during the maturation of the corresponding pre-mRNAs [7].

**Figure 1:**
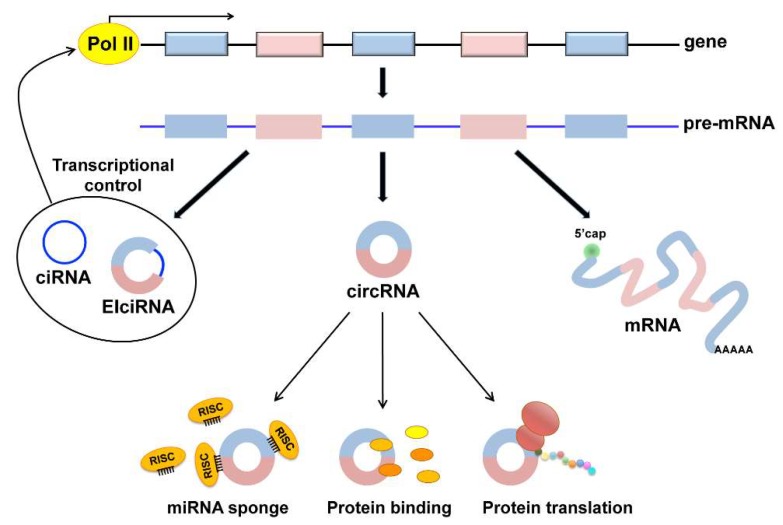
Putative functions of circRNAs. In addition to the production of messenger RNA (mRNA), back splicing events from the pre-mRNA molecules may lead to the formation of circRNAs. Three types of circRNAs have been identified, exonic (circRNAs), intronic (ciRNAs), and exon-intronic (EIciRNAs). ciRNAs and EIciRNAs have been shown to play a role in the modulation of the transcription of their parental genes (bold arrows). The most abundant exonic circRNAs have been implicated in sponging miRNAs associated with the RNA-induced silencing complex (RISC) through multiple miRNA binding sites in the sequestration of bound proteins and in protein translation through a cap-independent mechanism (narrow arrows).

Based on the components of exons and introns from the parental genes, circRNAs can be divided into three categories: exonic circRNAs (circRNA) that only contain back-spliced exons, circular intronic RNAs (ciRNA) that come from introns, and exon-intron circRNAs (EIciRNAs) formed by both exons and introns [8] (Figure 1). Most circRNAs are generated from the back-splicing of pre-mRNAs, in which downstream donor-exons splice to upstream acceptor-exons. Exonic circRNAs are the most currently studied circRNAs and are predominantly localized in the cytoplasm. EIciRNAs are formed by introns ‘retained’ between exons during the back-splicing process and are located mostly in the nucleus. CiRNA processing depends on consensus motifs containing 7 nucleotide GU-rich elements close to the 5′ splice site and 11 nucleotide C-rich elements proximal to the branch point site. Circular intronic RNAs contain a single circularized intron and accumulate in the nucleus to regulate the gene transcription of their parental genes through unknown mechanisms [9].

More than 80% of the circRNAs consist of protein-coding exons, whereas smaller fractions align with introns, long noncoding RNAs, unannotated regions of the genome and antisense regions to known transcripts [10]. The length of most exonic circRNAs is less than 1500 nt, the median length being ∼500 nt. Analysis of the number of circRNAs from their host genes revealed that one gene could produce multiple circRNAs, with about 50% of the host genes expressing one circRNA at significantly higher levels [10].

Because of their non-linear conformation and lack of polyadenylated (poly(A)) tails, very few circRNAs can be identified by the next-generation RNA sequencing (RNA-seq) profiling of poly(A)- RNAs, making many datasets generated in the past with different purposes not usable.

Conversely, circRNAs are readily detectable in ribosomal RNA-depleted RNA-seq datasets. Moreover, the use of the RNase R, which preferentially digests linear RNAs, allows us to enrich circRNAs, facilitating their detection.

A number of algorithms have been developed to detect global circRNA expression from different RNA-seq datasets by mapping reads to back-splice junctions [11,12,13,14,15,16,17,18]. The need to consider only the reads across the junctions enormously reduces the number of reads available for circRNA analysis, posing a variety of computational problems that are yet to be completely addressed [18,19]. Among the most popular circRNA detection tools, there are CIRI2 [20], CIRCexplorer [17], and KNIFE [21]. However, no single method excels over the others in terms of precision and sensitivity, stressing the need for further bioinformatics research. Thus, in order to obtain a reliable analysis, several algorithms should be combined [18,22,23], followed by experimental validation.

A thorough circRNAs validation requires several pieces of evidence, including confirmation of the presence of the back-splice junction by qPCR experiments using divergent primers. The resistance of the identified RNAs to RNase R exonuclease is also informative, although the specificity of this assay is limited [24].

Another crucial element for the advancement of the field is the availability of a comprehensive database that acts as an archive of circRNA sequences and annotations (reviewed in [25]). CircBase (http://www.circbase.org/) and circNet (http://circnet.mbc.nctu.edu.tw) are among the most widely used, but their manual curation slows down the inclusion of the increasing amount of studies published. So far, there are no comprehensive databases collecting all information on circRNAs that are generic, structured, updated frequently and partially automated, like the Gene Expression Omnibus [26], RefSeq [27], and miRBase [28].

For this reason, in this review, we will name circRNAs as originally indicated by the authors who identified them.

## 3. Circular RNA Expression and Function

Although circRNAs are generally expressed at low levels compared to mRNAs [12,13,14,16,29], some of them are abundant and expressed independently of their corresponding linear transcripts [17,29,30,31,32].

Expression of endogenous circRNAs is regulated at different levels. Following the Pol II transcription of the pre-mRNAs, cis- and trans-regulatory factors can influence the efficiency of back-splicing, including the presence of intronic complementary sequences, of core spliceosomal components, and of other regulatory RNA-binding proteins (RBPs). Circularization of transcribed exons may result from exon-skipping via a lariat structure or direct circularization driven by intronic pairing [13,16,33,34].

RBPs have been shown to regulate circRNA production both positively and negatively, mostly through their double strand RNA binding domain or through binding to specific RNA motifs [7]. One interesting example is the circMbl that is generated from the pre-mRNA of the splicing factor muscleblind (MBNL1). This circRNA and its flanking introns contain conserved MBNL1 binding sites and modulation of MBNL1 levels strongly affects the circMbl biosynthesis, suggesting that there is a feedback loop between MBNL1 and circMbl that regulates excess protein production [35].

The CircRNA turnover is also crucial for the regulation of their expression level. Although back-splicing is relatively inefficient, some circRNAs can accumulate themselves to high levels [36], likely because their circular structures are resistant to exonucleolytic degradation. Indeed, it is not yet clear how circular RNAs are eventually degraded. One proposed mechanism for circRNA clearance is through exosomes [37].

Many different functions have been hypothesized for circRNAs. They can regulate the transcription and splicing of their parental genes, can act as miRNA sponges, can regulate protein functions through the direct interaction circRNA/protein and can be translated into proteins with a cap-independent mechanism (Figure 1).

Because most circRNAs are derived from exons of protein-coding genes [16], their processing can affect the splicing of their precursor transcripts, leading to altered gene expression outcomes. Furthermore, nuclear localized circular RNAs can modulate gene expression at both the transcription and splicing levels [9,38,39].

Recent studies have shown that several abundant circRNAs can function as miRNA sponges. The best example is CDR1as (also known as CiRS-7), a single-exon, highly conserved, and abundant circRNA expressed at high levels in the mammalian brain [14,40]. CDR1as/CiRS-7 contains over 60 binding sites for miR-7, is localized in the cytoplasm, and both CDR1as/CiRS-7 and miR-7 are bound by the miRNA effector protein AGO2. CDR1as/CiRS-7 and miR-7 have an overlapping and specific expression in neuronal tissues and the knock-down of CDR1as/CiRS-7 triggers the downregulation of miR-7 targets, suggesting that CDR1as competes for miR-7 binding, acting as a miRNA-sponge [14,41]. Many other circRNAs have been found to contain miRNA binding sites, but their regulatory role on the bound miRNAs is still undetermined in many circumstances. Since the majority of mammalian circRNAs are expressed at low levels and rarely contain multiple binding sites for the same miRNAs, it is an object of debate on how relevant and frequent the role of circRNA as miRNA sponges may be [12,42]. Moreover, it is still unknown whether the binding of miRNAs to linear and circular RNAs occurs with exactly the same mechanism and whether RBPs might selectively facilitate the interaction between miRNAs and circRNAs. Indeed, circRNAs can interact with different proteins to form specific complexes. Again, the low abundance of circRNAs argues against a prominent regulatory role on protein function unless more circRNAs act synergistically on the same pathway.

In contrast to previous studies that failed to find evidence of translation from circRNAs [12], recent papers reported that a small subset of endogenous circRNAs is translatable through a cap-independent mechanism [43,44,45,46]. Specifically, the muscle-specific circRNA circ-ZNF609, sharing the start codon with the linear transcript, is associated with heavy polysomes and is translated into a protein in a splicing-dependent and cap-independent manner [43]. Two additional studies in flies and human cells reported that a fraction of circRNAs is found associated with polysomes and can be translated into proteins in a cap-independent manner [44]. Interestingly, circRNAs are enriched with methylated adenosine that is important to drive translation [46]. However, since both the proportion of polysome-associated circRNAs and the translation efficiency are low, not many proteins are expected to be produced by circRNAs at biologically relevant levels. It should also be mentioned that, while translation on circRNAs can be initiated, auxiliary factors are needed for the efficient elongation and termination [45].

Further studies are clearly needed to highlight the functional implications of circRNAs in normal biology and in disease. The available experimental evidence has shown that different circRNAs have specific mechanisms of action rather than general functions, and each one is required to be investigated in detail.

## 4. Circular RNAs Are Implicated in Both Physiology and Pathology

Recent data obtained from circRNA expression profiles in many different human tissues show that circRNAs are both spatially and temporally regulated, their expression patterns being cell- and tissue-specific [47,48]. Indeed, some circRNAs have been found to be more expressed than their linear isoforms, implicating a functional relevance [10,12,13,14,16,29]. These data suggest that they may play a role in the normal development of tissues or organs, in disease pathogenesis, and that they may be used as disease biomarkers. Evidence corroborating this indication is rapidly accumulating in different fields.

circRNAs have been extensively studied in neuronal tissue due to their abundant production from neural genes and high accumulation levels in neuronal cell lines in the mammalian brain and aging neural tissues in flies [31,32,35,36,49]. Accordingly, many circRNAs are upregulated during neural development compared to their linear isoforms [31,36] and circRNAs have also been implicated in neuronal diseases. For example, CDR1as/CiRS-7 is dramatically reduced in sporadic Alzheimer’s disease [50] and a group of circRNAs has been found to be regulated by the Fused in Sarcoma (FUS) RNA binding protein in an amyotrophic lateral sclerosis-associated cellular model [51].

CircRNAs have also been involved in cancer development. The deregulation of circRNAs may influence proliferative signaling, epithelial-to-mesenchymal transition, angiogenesis, apoptosis or drug resistance and, in this manner, have a direct causative role in the development of cancer [52].

In this review, we will focus on the role of circRNAs in the development and dysfunction of skeletal and cardiac muscles.

## 5. Circular RNAs in Myogenesis

### 5.1. Skeletal Muscle

The dystrophin gene (*DMD*), one of the largest known genes producing many different transcripts in skeletal muscle cells, was among the first genes where circRNAs were identified as RNA circles consisting of exons that were skipped by alternative splicing [53]. In this report, circular DMD transcripts were identified in normal skeletal muscles for the first time. In contrast to the previous idea that circRNAs might simply be functionless products of aberrant splicing events [54], the formation of circRNAs from the *DMD* gene was shown to be not necessarily linked to exon skipping, but to be regulated by “undetermined factors”, mainly because no tight correlation was found between the identified spliced transcripts and the circRNAs expected to be produced [53].

CircRNAs have been identified in numerous species, including nematodes, flies, birds, and mammals. Specifically, a recent RNA-seq analysis of circRNAs in striated muscle from different animals showed that they are abundant and dynamically expressed during muscle development and aging [55,56,57,58,59,60] (Table 1). As in other tissues, most circRNAs identified in skeletal muscles are exonic (80–90%) and several of them show differential expression between developmental stages [57,60] and different age conditions [55,57]. Many circRNAs have been predicted to interact with miRNAs [57,59]. In agreement with a putative regulatory role on muscle gene expression by circRNAs, gene ontology (GO) and KEGG enrichment analysis of the host genes of the identified circRNAs showed that they were mainly involved in muscle-related biological processes [58,59].

The analysis of differentially expressed circRNAs during myogenic differentiation was also performed in cultured myogenic cells, both in C2C12 murine myoblasts and in primary human myoblasts [43,61,62] (Table 1).

In one study [62], deep RNA-seq of C2C12 myoblasts during cell differentiation revealed 37,751 unique circRNAs derived from 6943 hosting genes. A few randomly chosen circRNAs were validated by qPCR and RNA fluorescence in situ hybridization. Bioinformatics analysis showed dynamic circRNA expression changes during myoblast differentiation, the circRNAs abundance being independent from that of their cognate linear RNAs. Gene ontology of the host genes showed that many down-regulated circRNAs were related to the cell division and cell cycle, whereas up-regulated circRNAs were associated with the cell development process. Bioinformatics analysis of potential circRNA-miRNA interactions identified miRNAs involved in the regulation of myoblast differentiation, such as miR-133, miR-24, and miR-23 [62]. In another investigation [61], expression profiles of C2C12 myoblasts and myotubes were assessed using microarrays. Over 11,000 circRNAs were detected, 581 of which were differentially regulated. Additionally, in this case, bioinformatics analysis of relevant linear transcripts and of potential circRNA-miRNA interactions indicated the association of the identified circRNAs to myogenesis. Interestingly, 224 circRNAs were predicted to have a coding potential based on the number of open reading frames and N6-methyladenosine motifs [61]. Both these studies indicate an implication of the identified circRNAs in the myogenic process, stimulating the experimental validation of the suggested mechanisms.

As shown in Table 1, the numbers of circRNAs identified in the reported studies are very different, ranging from a few hundred to several thousand. It should be mentioned that such a variability is linked to several factors: the enrichment for circRNAs through RNAse R treatment, RNA-seq depth, and the filters applied for selections.

A parallel expression profiling of proliferating and differentiated human primary myoblasts and mouse (C2C12) myoblasts revealed a global change of circRNA expression [43]. Notably, circRNA abundance and circular to linear ratio generally increased during differentiation, as also observed upon neuronal differentiation [31], possibly related to the high stability of circRNAs accumulating in terminally differentiated cells over time. All circular species were almost exclusively located in the cytoplasm, both in mouse and human cells. A subset of 17 conserved circRNAs was selected according to their expression level, modulation during differentiation, and circular/linear ratio. Next, a high-content functional genomics screening was used to establish how each of these circRNAs affected cell differentiation and proliferation using a dedicated circRNA knockdown strategy followed by a functional screening. Circ-QKI and circ-BNC2 emerged to have promoting and inhibitory effects on differentiation, respectively, while circ-ZNF609 regulated myoblast proliferation (Table 2).

Further studies on circ-ZNF609 provided an interesting example of a protein-coding circRNA. Circ-ZNF609 was shown to be associated with polysomes, although at low levels, and to be translated into a protein in a splicing-dependent and cap-independent manner when ectopically expressed [43]. Interestingly, *Zfp609*, the murine homolog of ZNF609, contains miRNA binding sites and was recently shown to inhibit the expression of myogenic transcription factors in C2C12 myoblasts possibly by sponging miR-194-5p [69] (Table 2).

#### circRNAs as miRNA Sponges in Skeletal Muscles

In agreement with the finding that many differentially expressed circRNAs identified in profiling of muscle cells/tissues have been predicted to sponge miRNAs, a number of recent studies have described specific circRNAs that could regulate the miRNA expression by competitive binding (Table 2). CircSVIL, previously shown to be highly expressed during late embryonic development in chicken [59], carries 4 binding sites for miR-203 and was recently reported to interact with miR-203 by a dual-luciferase reporter and RNA pull-down assays and to increase the expression of miR-203 target genes *c-JUN* and *MEF2C*. The inhibition or overexpression of circSVIL in chicken myoblasts showed that it promoted myogenic differentiation, possibly through sponging miR-203 [59].

Other circRNAs, identified as differentially expressed by comparing adult to embryonic bovine muscle tissue, were shown to regulate differentiation in bovine myoblasts: circFGFR4 by binding miR-107 could promote cell differentiation via targeting Wnt3a [70]; circFUT10 was shown to accelerate differentiation and decrease the proliferation of myoblasts by inhibiting the miR-133a activity [71]; circLMO7 was shown to inhibit bovine myoblast differentiation, promote cell proliferation, and protect myoblasts from apoptosis by binding miR-378a-3p [60]. While circFGFR4 contained 18 putative miR-107 binding sites, circFUT10 and circLMO7 contained only three and one miRNA binding sites, respectively. These studies highlight a potential regulatory role of circRNAs in the myogenesis via the sequestration of miRNAs. As noted before, however, functional interactions between endogenous miRNAs and circRNAs need to be validated since the number of miRNA binding sites identified in the circRNA sequences is very low in some cases and the use of circRNA-miRNA overexpression for functional studies might be misleading.

### 5.2. Cardiac Muscle

Little is known about the regulation and role of circRNAs in normal heart development, and most information is derived from hESC (human embryonic stem cells) differentiated towards cardiomyocytes (CMs) (Table 1).

By studying the modulation of circRNAs during human fetal development, Szabo and collaborators identified back-splicing events during weeks 10–20 of fetal heart development [21], and found that the levels of one of the most abundant circRNAs, circular NCX1, increased more rapidly than the expression of its host gene. Moreover, both the circular RNAs NCX1 and RHOBTB3 were induced during in vitro directed differentiation of human embryonic stem cells to CMs [21]. The datasets generated in this study were further analyzed by Li et al., integrating the changes in the expression of lncRNAs, circRNAs, and protein-coding genes at sequential stages of heart differentiation [63]. This analysis allowed for the identification of 198 modulated circRNAs. Most of them (161 out of 198) were differentially expressed during the progression from precursor cells to CMs, unlike lncRNAs and mRNAs that changed mostly during earlier stages of differentiation. An enrichment of circRNAs in differentiated CMs compared to undifferentiated stem cells was also confirmed in an independent study comparing human induced pluripotent stem cells (hiPSCs) and hiPSC-derived CMs [64].

Tan and collaborators [65] also used the hESC at different stages of differentiation (precardiac mesodermal progenitors, cardiac progenitors, immature CMs) and found thousands of exonic circRNA across five time-points. In particular, 479 circRNAs were strongly correlated to the differentiation time-course and a gene ontology analysis indicated that their host genes were significantly enriched in terms related to heart development [65]. Intriguingly, Titin (*Ttn*) circRNA isoforms increased as the cell differentiation progressed towards CMs. At the end of the differentiation time-course, the majority of the *Ttn* circRNA isoforms were derived from the sarcomeric isotropic (I)-band, as observed in adult human hearts [65]. A similarly high dynamic of *Ttn*-derived circRNAs was observed by comparing neonatal and adult rat hearts [67].

A separate study, also using hESC at various differentiation stages as a model, identified thousands of circRNAs with host gene-independent expression; interestingly, the host genes of 320 of them were related to cardiac development. In the same study, a subset of rodent homologs of circMYOD, circSLC8A1, circATXN7, and circPHF21A was shown to interact with ribosomes and/or AGO2 protein complexes, suggesting a role for these circRNAs in the regulation of RNA translation [66] (Table 3).

## 6. Circular RNAs in Muscle Diseases

Alternative splicing is tightly regulated in different tissues and developmental stages and its disruption can lead to a wide range of human disorders. Striated muscles are one of the tissues with the highest number of differentially expressed alternative exons and disruption of normal splicing has been variously implicated in the pathophysiology of muscle disease [72]. Indeed, high expression levels of circRNAs were detected in normal muscle tissue (see section above) and evidence of circRNAs dysregulation in muscle diseases is emerging.

### 6.1. Skeletal Muscle Diseases

Duchenne muscular dystrophy (DMD), which involves a progressive deterioration of muscle function, is caused by frame-shifting deletions or nonsense mutations in the *DMD* gene resulting in the absence or reduced production of the dystrophin protein [73]. As described above, circRNAs produced by spliced transcripts from the *DMD* gene were among the first to be identified in skeletal muscles. These circRNAs were generated mostly at the 5’ end of the transcript [53]. A recent paper focused on the characterization of circRNA production in the region spanning exon 45 to exon 55 of the *DMD* gene that represents a deletion hotspot in 63% of DMD patients [74]. Interestingly, it has been shown that multiple exon skipping, targeting exon 45–55, might improve patients’ symptoms because patients who have a genomic deletion of all these exons show very mild symptoms. The authors identified the preferential splice-sites involved in both circRNA generation and in multiple exon skipping of exon 45–55. Results obtained corroborate a circRNA-generation model in which the interaction between upstream and downstream introns triggers multiple exon skipping and generates circRNAs [74].

Recently, RNA-seq data from both normal and dystrophic human myoblasts derived from DMD patients were analyzed. Hierarchical clustering analysis of normal and dystrophic myoblasts and myotubes revealed that indeed DMD cells have a unique signature in terms of circRNA expression levels: specific subsets of transcripts were differently abundant in DMD patient-derived myoblasts and myotubes [43]. Interestingly, both circ-QKI and circ-BNC2 circRNAs, upregulated during in vitro differentiation of normal myoblasts as described above, were downregulated in the DMD conditions, well correlating with the notion that dystrophic cells have altered progression into the differentiation process [75]. Conversely, the protein-coding circ-ZNF609, which was downregulated during myogenesis in control myoblasts, was found expressed at elevated levels in differentiated DMD myoblasts [43].

Overall, these observations suggest that circRNAs may play a functional role in DMD and could potentially be exploited as therapeutic tools.

Many other muscular dystrophies show splicing alterations [76]. Identification and characterization of the circRNAs abundantly expressed in these splicing-related diseases are urgently needed in order to highlight their role in pathogenetic mechanisms and to develop future therapies.

### 6.2. Cardiac Muscle Diseases

Cardiovascular diseases are the most serious health problem worldwide, being the diseases with the worst rate of morbidity and mortality (http://www.who.int/mediacentre/factsheets/fs317/en/). Specifically, the molecular mechanisms of heart diseases are still known only in part and the role of circRNAs has just started to be investigated.

Through RNA-seq, Khan and collaborators [77] identified a subset of circRNAs deregulated in dilated cardiomyopathy (DCM) and/or in hypertrophic cardiomyopathy (HCM). Interestingly, they found that, of the 80 circRNAs produced from the *Ttn* gene, some were dynamically regulated in DCM but not in HCM and their expression was dependent on the splicing factor RBM20, linking together the RBM20 function, circRNAs, and myocardial disease development [77].

Another inventory of cardiac circRNAs is present in the study of Werfel et al., which profiled by RNA-seq analysis circRNAs expressed in human heart failure patients and in a mouse model of cardiac hypertrophy [67]. Again, dozens of circRNAs arising from the *Ttn* gene were identified.

A list of the studies on circRNA profiling is shown in Table 1.

In the following paragraphs, recent emerging studies on circRNAs involved in cardiomyopathies (Table 3) are described and categorized according to their mechanism of action.

#### 6.2.1. circRNAs as miRNA Sponges in Heart Disease

As described above (Section 3), circRNAs can act as miRNA sponges, although many criteria need to be satisfied in order to provide solid evidence for this mechanism of action.

The prototype of this group of circRNAs is CDR1as/CiRS-7 that has been shown to interact with miR-7 in mouse brain tissue, counteracting miR-7 function [14,41]. CDR1as/CiRS-7 has been found to also play a role in the heart, increasing the stress-induced apoptosis in CMs and increasing the infarct size in a mouse model of myocardial infarction (MI) [78]. Data obtained in vitro suggest that the upregulation of CDR1as/CiRS-7 induced by hypoxia in CMs reduces the miR-7 bioavailability and de-represses its targets by sequestering the miRNA.

Another example is provided by mm9-circ-012559/HRCR (heart-related circRNA), the expression of which has been found to be reduced in mouse hypertrophy and failure [79]. Indeed, mm9-circ-012559/HRCR can bind miR-233, counteracting its repressor activity on ARC (Apoptosis repressor with CARD domain), which in turn protects the mouse heart from dysfunction.

In an independent study, the link between a circRNA, mm9-circ-016597/MFACR (mitochondrial fission and apoptosis-related circular RNA), a miRNA, miR-652-3p, and its target MTP18 was established, showing that mm9-circ-016597/MFACR induces mitochondrial fission and apoptosis, aggravating MI injury [82].

The presence of diabetes mellitus, which is responsible for a complex scenario of biochemical, functional, and morphological alterations in CMs, significantly increases the risk of developing diabetic cardiomyopathy, which can cause heart fibrosis and remodeling even in the absence of atherosclerosis, hypertension and other cardiac pathologies [85]. Tang et al. [80] found that the expression of circRNA_000203 is elevated in both Angiotensin II treated cardiac fibroblasts and in the heart of diabetic mice. The function of circRNA_000203 was studied in mouse cardiac fibroblasts where it was found that circRNA_000203, by sequestering miR-26b-5p, de-represses its targets *Col1a2* and Ctgf, indicating a pro-fibrotic function.

Additionally important for cardiac fibrosis seems to be circRNA_010567, which is markedly increased in diabetic hearts [81] as well as in a mouse model of cardiac hypertrophy [68]. In vitro experiments indicate that circRNA_010567 displays a pro-fibrotic function by sequestering miR-141 and by increasing the transcription of its target TGF-β1 [81].

#### 6.2.2. circRNAs Interacting with RNA-Binding Proteins in Heart Disease

Another mechanism of action of circRNAs is via the binding of RBPs.

Mouse treatment with the chemotherapy drug doxorubicin induces myocardial dysfunction triggering oxidative stress [86]. This condition is associated with an increase of circ-Foxo3 levels that, in turn, contributes to heart function deterioration, dilation, and fibrosis [83]. Circ-Foxo3 can act by sequestering in the cytoplasm different senescence—(ID1 and E2F1) and stress—(HIF1a and FAK) associated RBPs, preventing their protective functions and resulting in an increased cellular senescence.

Conversely, circ-Amotl1 has a protective effect in doxorubicin-induced cardiomyopathy [19]. Mechanistically, in vitro experiments showed that circAmotl1 is able to induce AKT (AKT Serine/Threonine Kinase 1) phosphorylation and nuclear translocation, leading to enhanced cell proliferation and survival [19].

Another protein important for circRNA function is Quaking (QKI). This RBP can regulate circRNA expression during epithelial–mesenchymal transition [30]. The treatment of mouse CMs with doxorubicin is associated with reduced levels of Qki protein and of circRNAs derived from *Ttn* [84]. Specifically, silencing of circRNAs derived from the 105–111 region of *Ttn* increased the apoptotic effect of doxorubicin in vitro, while the overexpression of Qki5, the most abundant cardiac isoform of Qki, in mouse hearts reduced the doxorubicin-induced cardiac apoptosis and atrophy, and improved cardiac function [84]. These findings suggest that *Qki5* and *Ttn* 105–111 derived circRNAs may play a cardioprotective role upon its exposure to cardiotoxic drugs.

## 7. Therapeutic Perspectives of circRNAs

**Figure 2:**
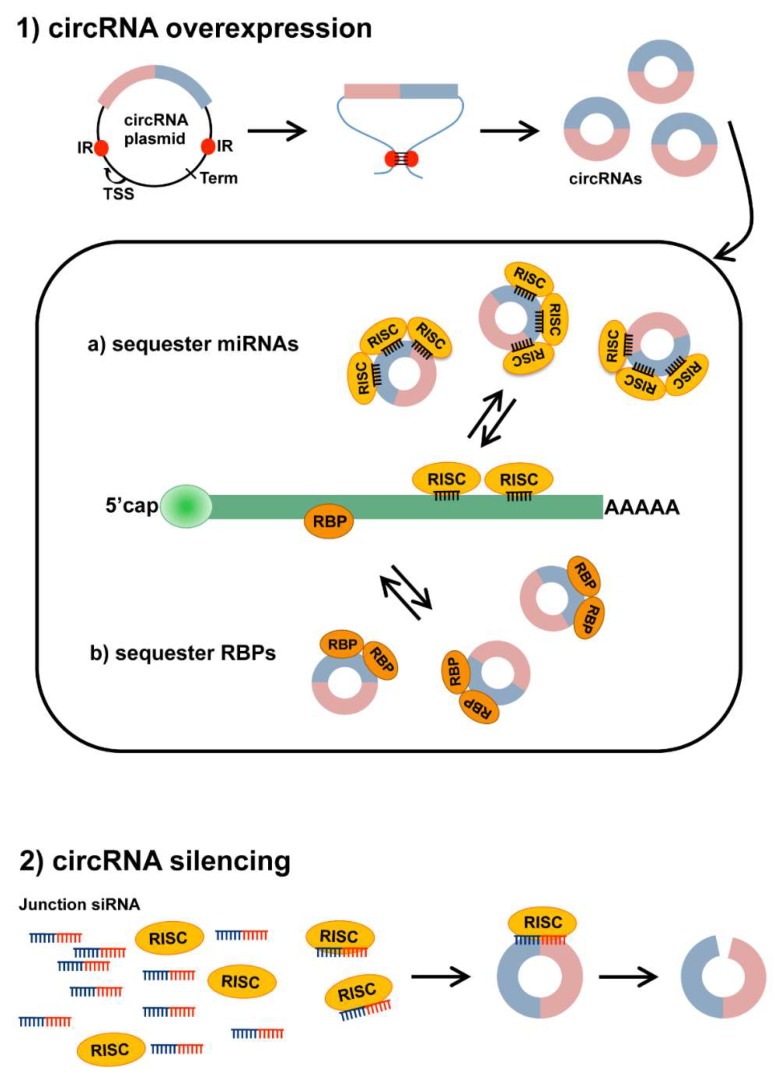
Strategies to modulate circRNA expression. (**1**) Plasmids designed to overexpress circRNAs contain the circRNA sequence (colored boxes) flanked by splicing signals (not shown) and intronic sequences including inverted repeats (IR, red dots). IR pairing within the transcribed RNA induces the formation of a secondary structure that facilitates back splicing and circularization. TSS: Transcriptional Start Site; Term: Transcriptional Terminator. Produced circRNAs can then (**a**) sequester miRNA-RISC complexes to inhibit miRNA action on mRNA targets or (**b**) interact with RNA-binding proteins (RBPs), sequestering them and preventing their activity; (**2**) silencing of circRNAs can be achieved through treatment with a siRNA complementary to the back-splice junction sequence (Junction siRNA). This should allow us to target specifically the circRNA, but not the corresponding linear transcript. The siRNA, loaded on the RISC complex, can selectively bind the circRNA and induce its degradation by endonucleolytic cleavage.

As described in the previous sections, the evidence is rapidly accumulating supporting a prominent role of circRNA in the development of both skeletal muscles and cardiac diseases. To date, no clinical trials have exploited circRNAs as therapeutic tools or as targets in the cardiac or skeletal muscles, but this scenario will likely change in the future (Figure 2).

When designing circRNA therapeutics, there are some important issues to be considered, most of which are in common with all gene-based therapies [87].

The overexpression of exogenous circRNAs for gene therapy can be obtained by inserting DNA cassettes designed for circRNA expression in specific vectors and delivering them to the muscle. These vectors contain the circRNA sequence flanked by introns bearing inverted repeats and the necessary splicing signals to facilitate circularization [7,8,18,88]. CircRNAs can also be synthesized in the lab and delivered directly to target tissues [89]. Exogenous circRNAs can act as miRNA or RBP sponges, sequestering specific miRNAs/RBP [90] or can be engineered to allow a robust and stable protein expression [89]. CircRNAs might be superior to linear RNAs for therapeutic purposes in vivo, as their endogenous stability may be higher, and additional covalent modification of their backbone may increase their use even further. However, care should be taken not to induce an interferon response by the target cells, triggered by the introduction of foreign circRNA [91].

The inhibition of undesired circRNAs should be carried out in a manner that does not interfere with the expression of the corresponding linear mRNA [7,8,18]. The most straightforward strategy is to target the unique back-splice junction of the relevant circRNA by the exogenous delivery of specific siRNAs. A possible alternative is interfering with the back-splicing process administrating oligonucleotides complementary to sequences necessary for back-splicing in the pre-mRNA, such as flanking intronic Alu repeats or binding sites for RBPs stimulating back splicing.

Finally, circRNAs have a strong biomarker potential, being readily detectable in the peripheral blood and in extracellular vesicles present in body fluids [92]. For instance, it has been shown that the circRNA MICRA is associated with heart failure after acute myocardial infarction, allowing us to predict the development of functional failure and enabling risk stratification [93,94].

## 8. Conclusions

Although only recently (re)discovered, circRNAs are rapidly entering the spotlight, attracting the attention of an increasing number of researchers, as circRNA’s role in a variety of cellular functions and pathways emerges. Nevertheless, our knowledge of circRNA biology is still very rudimentary, while a detailed understanding of their biogenesis, localization, and function is necessary before trying to find a clinical application for these new molecules.

Indeed, there are many issues requesting closer attention and inviting caution. For instance, while the number of circRNAs detected in different tissues in physiological and pathological conditions is increasing exponentially, most of them seem to be expressed at very low levels [29,95] and may indeed likely represent just a byproduct of pre-mRNA maturation.

Many papers indicating that circRNAs play a role in muscle biology or muscle diseases, suggest that circRNAs act by miRNA sequestration. However, the circRNA role as miRNA sponges is probably overestimated [12,42], possibly because software predicting miRNA binding are available, facilitating the exploration of this molecular mechanism. A more plausible hypothesis is that there is not a main mechanism of action and that each circRNA needs to be studied individually, as observed for proteins.

Another crucial issue hindering the progression of the field is the lack of an internationally recognized nomenclature and of a unique database, such as miRBase for miRNAs.

In spite of these challenges, with the current pace of discoveries, we can anticipate a rapid progression in deciphering the basic mechanisms of circRNA functions and an emerging role in the near future for circRNA-based diagnostic or therapeutic strategies for skeletal and cardiac muscle disorders.

## Figures and Tables

**Table 1:** The CircRNA expression of profiling studies in skeletal and cardiac muscles.

Sample	Organism	Experimental Conditions	Method	Detected circRNAs	qPCR Validated	Ref
Skeletal muscle						
primary myoblasts	Homo sapiens	GM vs. DM	RNA-seq	2100	29	[43]
vastus lateralis	Macaca mulatta	Aging	RNA-seq	12,000	8	[55]
C2C12 cell line	Mus musculus	GM vs. DM	RNA-seq	1600	29	[43]
C2C12 cell line	Mus musculus	GM vs. DM	MicroArray	11,000	8	[61]
C2C12 cell line	Mus musculus	GM vs. DM	RNA-seq	37,700	10	[62]
longissimus dorsi	Sus scrofa	Aging	RNA-seq	4400	2	[57]
longissimus dorsi	Ovis aries	Embryo vs. Adult	RNA-seq	6000	10	[58]
longissimus dorsi	Ovis aries	Adult skeletal muscle	RNA-seq	886	9	[56]
longissimus dorsi	Bos taurus	Embryo vs. Adult	RNA-seq	13,000	17	[60]
leg muscles	Gallus gallus	Embryo development	RNA-seq	13,400	6	[59]
Cardiac muscle						
Embryonic Stem Cells	Homo sapiens	Differentiation to adult cardiomyocytes	RNA-seq	1702	ND	[63]
Embryonic Stem Cells	Homo sapiens	Differentiation to adult cardiomyocytes	RNA-seq	5602	9	[64]
Embryonic Stem Cells	Homo sapiens	Differentiation to adult cardiomyocytes	RNA-seq	6853	6	[65]
Embryonic Stem Cells	Homo sapiens	Differentiation to adult cardiomyocytes	RNA-seq	4518	2	[66]
heart	Rattus rattus	Neonatal vs. Adult	RNA-seq	>9000	6	[67]
left ventricle	Mus musculus	Sham vs. TAC	MicroArray	1163	20	[68]
left ventricle	Homo sapiens	Failured vs. healthy	RNA-seq	1363	ND	[67]
left ventricle	Mus musculus	Sham vs. TAC	RNA-seq	675	ND	[67]

Abbreviations: GM = growth medium; DM = differentiation medium; TAC = transverse aortic constriction; RNA-seq = RNA-sequencing; ND = not determined.

**Table 2:** The CircRNAs identified and characterized in the models of skeletal muscles.

circRNA	Organism	Molecular Function	miRNA Binding Site	Biological Role	Experimental Approach	Ref
circ-ZNF609	Homo sapiens	Protein encoding	NA	Promotes myoblast proliferation	Inhibition of circRNA	[43]
circ-QKI	Homo sapiens	ND	ND	Induces differentiation	Inhibition of circRNA	[43]
circ-BNC2	Homo sapiens	ND	ND	Induces differentiation	Inhibition of circRNA	[43]
circ-Zfp609	Mus musculus	Protein encoding	NA	Promotes myoblast proliferation	Inhibition of circRNA	[43]
circ-Zfp609	Mus musculus	microRNA binding (miR-194-5p)	4	Inhibits differentiation	Inhibition/Overexpression of circRNALuciferase assay	[69]
circFGFR4	Bos taurus	microRNA binding (miR-107)	18	Promotes myoblast differentiation and apoptosis	Overexpression of circRNA RNA pulldown Luciferase assay	[70]
circLMO7	Bos taurus	microRNA binding (miR-378a-3p)	1	Promotes myoblast proliferation and inhibits differentiation and cell apoptosis	Overexpression of circRNA Luciferase assay	[60]
circFUT10	Bos taurus	microRNA binding (miR-133a)	3	Inhibits myoblast proliferation and induces differentiation and cell apoptosis	Overexpression of circRNA Luciferase assay	[71]
circSVIL	Gallus gallus	microRNA binding (miR-203)	4	Promotes myoblast proliferation and differentiation	Inhibition/Overexpression of circRNA Luciferase assay RNA pull-down RISC-IP	[59]

Abbreviations: NA = not applicable; ND = not determined; RISC-IP = RNA-induced silencing complex-immunoprecipitation.

**Table 3:** The CircRNAs identified and characterized in models of healthy and diseased cardiac muscles.

circRNA	Organism	Molecular Function	miRNA Binding Site	Biological Role	Experimental Approach	Ref
CDR1/CIRS7	Mus musculus	microRNA binding (miR-7a)	70	Induces apoptosis and worsens MI injury	Overexpression of circRNA	[78]
mm9-circ-012559/HRCR	Mus musculus	microRNA binding (miR-223)	6	Protects the heart from hypertrophy and failure	RNA pull-down/Overexpression of circRNA	[79]
circMYOD	Mus musculus	Ribosome interaction	NA	Associated to cardiac development	RiboTag RIP-seq	[66]
circSLC8A1	Mus musculus	Ago2-association	NA	Associated to cardiac development	Argonate RIP	[66]
circATXN7	Mus musculus	Ribosome interaction	NA	Associated to cardiac development	RiboTag RIP-seq	[66]
CircPHF21A	Mus musculus	Ago2-association	NA	Associated to cardiac development	Argonate RIP	[66]
circRNA_000203	Mus musculus	microRNA binding (miR-26b-5p)	2	Induces fibrosis	RNA pull-down/Overexpression of circRNA	[80]
circRNA_010567	Mus musculus	microRNA binding (miR-141)	ND	Induces fibrosis	Inhibition of circRNA	[81]
mm9-circ-016597/MFACR	Mus musculus	microRNA binding (miR-652-3p)	15	Induces mitochondrial fission and apoptosis, aggravates MI	Inhibition/Overexpression of circRNA	[82]
circ-Foxo3	Mus musculus	RNA-binding protein interaction (ID1, E2F1, HIF1a, FAK)	NA	Induces cellular senescence; detrimental for cardiac function	Inhibition/Overexpression of circRNA RNA pull-down	[83]
circ-Amotl1	Mus musculus	RNA binding protein interaction (AKT, PDK)	NA	Stimulates cell proliferation and survival; cardioprotective	Inhibition/Overexpression of circRNA RNA pull-down	[19]
circTtn 105–111	Mus musculus	RNA binding protein interaction (Qki5)	NA	Protects from doxorubicin-induced apoptosis	Inhibition/Overexpression of Qki	[84]

Abbreviations: NA = not applicable; ND = not determined; RIP = RNA-immunoprecipitation.
